# The motivational potential of meaningful work: Relationships with strengths use, work engagement, and performance

**DOI:** 10.1371/journal.pone.0197599

**Published:** 2018-06-13

**Authors:** Jessica Van Wingerden, Joost Van der Stoep

**Affiliations:** 1 Schouten Global, Centre of Research, Knowledge and Innovation, Zaltbommel, The Netherlands; 2 Erasmus University Rotterdam, Institute of Psychology, Rotterdam, The Netherlands; 3 VU University, Amsterdam, The Netherlands; Universita degli Studi di Firenze, ITALY

## Abstract

Research in the field of work and organizational psychology increasingly highlights the importance of meaningful work. Adding to this growing body of research, this study examined the complex linkage between meaningful work and performance. More specifically, we hypothesized that meaningful work has a positive relationship with an employee’s performance in several and interrelated ways, via employees’ use of strengths, via work engagement, and via strengths use affecting work engagement. We conducted a structural equation modeling on a sample of 459 professionals working at a global operating organization for health technology. The results provided support for the proposed model which showed a better fit than the sequential mediation model and the direct effects model. This indicates that the meaningful work–performance relationship is predicted best by multiple pathways via employees’ use of strengths and work engagement. The main theoretical, practical, and methodological implications of the results are discussed.

## Introduction

Meaningful work–defined as work that is experienced as particularly significant and holding positive meaning for an individual [1]—is a highly valued job characteristic by employees [2,3]. Some employees even value meaningful work above other work characteristics like income, job security, promotions, or working hours [4]. Experiencing meaningful work reflects a deep personal linkage between an employee and his or her work which motivates an employee to go above and beyond the normal requirements of their work [5]. A growing body of research links meaningful work to employee well-being and performance [6,7], and meaningless work to disengagement and alienation [8,9]. Due to these consequences, scholars and practitioners in the field of work and organizational psychology have been highly motivated to increase our understanding of the role that meaningful work plays within contemporary organizations.

However, despite the growing interest in the topic of meaningful work, hitherto relatively little is known about the processes through which meaningful work actually affects engagement and performance at work. Research in the positive psychology demonstrates that people are only able to excel when they use their personal strengths [10–13]. Here, strengths are defined as a natural capacity to behave, think and feel in a way that allows optimal functioning and performance in the pursuit of valued outcomes [14]. Because work is only perceived as meaningful when the outcomes are valued by the employee, we posit that meaningful work stimulates employees to use their strengths at work. This in turn affects performance directly and also indirectly via increased levels of work engagement. All in all, we present and test a meaningful work and performance model that specifies the intricacies of the relation between meaningful work and employee performance; and the mediating effects of strengths use and work engagement.

## Theory and hypotheses development

### The motivational potential of meaningful work

An employee experiences his or her work as meaningful when the work’s objectives are in line with his or her own ideals or standards [15]. Such an experience thus emerges when an employee’s personal beliefs, values and behaviors fit the specific requirements of work [16,17]. Whether or not employees perceive their work as meaningful primarily depends on the subjective interpretation of work and less on the objective reality [15]. In other words, the psychological meaningfulness of work represents the cognitive valuation of work as significant and meaningful by an employee. Although people vary in their actual perception of meaningful work because they differ in personality, every employee values the meaningfulness of his or her work to some degree (e.g., calling orientation, [18]).

The experience of meaningful work by employees positively affects personal and work-related outcomes [19–22]. For example, research demonstrated that employees who perceive their work as meaningful are, among other things, more committed to the organization and less likely to leave the organization. They are also more engaged and more productive than employees who do not consider their work as particularly meaningful [23–26]. Even more so, the experience of a lack of meaningful work has been linked to negative outcomes like increased cynicism [27,23]. Although the merits of experiencing meaningful work is clear, little is known of the processes through which meaningful work actually influences these positive or negative outcomes.

An answer could lie in the motivational potential that comes with doing meaningful work. Decades of research on employee empowerment has incessantly underlined the importance of meaningful work and its impact on an employee’s level of intrinsic motivation [15,28,29]. The perception of work as meaningful, in combination with a sense of self-efficacy, self-determination, and perceived impact lead to feelings of psychological empowerment, which subsequently triggers proactive behaviors [5,30,31]. For example, meaningful work has been associated with organizational citizenship behaviors, which are behaviors that go beyond formal role requirements (e.g. volunteering to do nonrequired tasks). This is because the experience of meaningful work fosters a sense of identification and involvement in the work place [5]. In other words, when employees feel that they do meaningful work, they feel connected with their work and with the outcomes of their work. This reflects the motivational potential of meaningful work which potentially explains the meaningful work–performance relationship. Below we will elaborate on this complex relationship in more detail and explore the mediating role of strengths use and work engagement in this relation.

### Increasing performance via strengths use and work engagement

Research in work and organizational psychology has shown that the use of strengths is associated with both increased engagement and performance [32,33]. Strengths use is a recurring concept in the positive psychology as the use of strengths has been repeatedly associated with sustainable well-being [11]. Upfront, it is important to differentiate between the possession and the actual use of strengths [34,35]. Strengths are individual characteristics, traits, and abilities that, when engaged, energize and allow a person to perform at his personal best [14,35]. Every person has certain strengths, regardless of whether others possess (more of) these strengths as well. The use or deployments of these strengths help people to perform at their best. For this reason, it is crucial to understand the factors in the work environment that motivate employees to use and capitalize their personal strengths at work.

How employees perceive their work is an important predictor of how they behave at work [36]. This cognitive assessment of the work environment–that is the experience of the work as meaningful–thus might also determine whether or not employees are motivated to use their strengths at work. Van Woerkom and colleagues (2016) demonstrated that the perception of organizational support predicts the use of strengths within the organization. This means that the subjective perception of organizational support determines whether or not the employee actually uses and capitalizes his or her personal strengths at work. In line with this reasoning, we argue that the motivation of employees to use their strengths at work largely depends on the experience of meaningful work–as meaningful work is highly valued by the employee and people tend to deploy their personal strengths in order to attain the valued objectives [14]. Therefore, we expect that the positive relation between meaningful work and performance is mediated by strengths use.

Besides strengths use, the level of work engagement by an employee is an important predictor of performance at work [37]. Even more so, work engagement–defined as “the positive, fulfilling and work-related state of mind that is characterized by vigor, dedication, and absorption” [38]–is a consequence of meaningful work because meaningful work starts a motivational process as it ignites a fire within the employee which leads to higher levels of work engagement [7,24]. Subsequently, higher levels of work engagement have been linked to increased performance. For this reason, we expect that the positive relation between meaningful and performance is mediated by work engagement.

**Fig 1:**
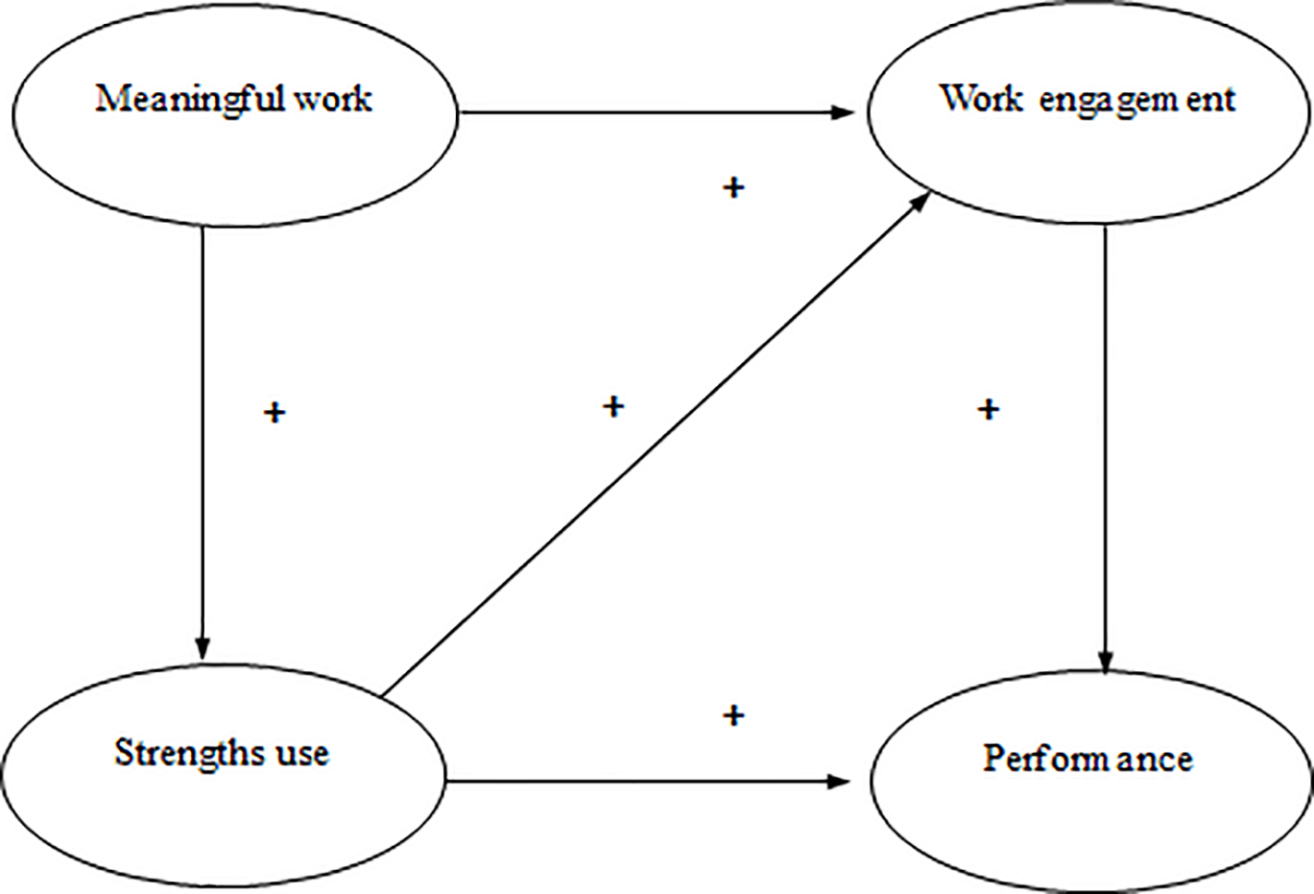
The meaningful work and performance model.

Additionally, we pose that meaningful work–performance relation is even more complex as we expect that meaningful work affects performance indirectly, via strengths use which subsequently triggers work engagement. This is because the use of strengths makes people feel authentic and efficacious and these positive psychological states fuel work engagement [34]. In other words, doing meaningful work motivates employees to use their strengths. This subsequently triggers a sense of efficacy and authenticity which fuels work engagement, which then affects performance at work. All in all, we propose a complex model which links meaningful work with performance in multiple ways a.) via strengths use, b.) via work engagement, c.) via strengths use which subsequently affects work engagement. All things considered, we propose and test a model that links meaningful work with performance among employees working at an organization for health technology (see Fig 1).

In order to better understand this process, consider teachers working in an educational setting. When they perceive their work as meaningful they truly value the outcomes of their work. This triggers different and interrelated processes which in the end positively determine their performance. A valued outcome in an educational setting could be, for example, the actual learning and personal development of students. As teachers value these outcomes they are more motivated to use their strengths. For example, some teachers possess the strength to deliver complex material in understandable chunks of information, while others are able to develop strong interpersonal relations with their students which encourages them to actively invest in their personal development. When teachers perceive their work as meaningful they are more motivated to capitalize their strengths and more engaged with their work. Both processes increase the likelihood that the teacher achieves the valued outcomes. Furthermore, teachers who use their strengths feel that they are more effective in their work and this increased level of self-efficacy further impacts their levels of work engagement. This in turn positively influences their performance. So all in all, the experience of meaningful work by teachers positively influences performance in several ways; via strengths use, via work engagement, and via strengths use affecting work engagement.

## Methods

### Procedure and participants

The sample consisted of 459 professionals working at a global operating organization for health technology. Of the 459 participants, 85% were male. The mean age of the participants was 42 years (*SD* = 10.58), and 84% had successfully finished a higher vocational education or university education in technology. The respondents participated voluntarily and did not receive any compensation for their contribution. The questionnaires were identical for all participants. The organization allowed the participants to fill in the questionnaires during their workday. The managing director introduced the online questionnaire in an email containing instructions and an explanation of the procedure, while also addressing the anonymity of the data. The online questionnaires were hosted by Schouten Global, and the managing director did not receive any information about individual outcomes. Furthermore, the data has been collected in accordance with the ethical guidelines of the American Psychological Association and the Dutch Association of Psychologists. As such, (1) participation was completely voluntary, (2) data collection through a self-report survey is exempted from an institutional ethics committee’s approval, and (3) the respondents did not receive any monetary compensation for their contribution. Informed consent was given by clicking on the “Finish” button on the last page of the survey.

### Measures

*Meaningful work* was measured using the Positive Meaning subscale of the Work And Meaning Inventory(WAMI; [39]). All 4 items were scored on a five-point Likert scale ranging from 1 (absolutely untrue) to 5 (absolutely true). Positive meaning (PM) was assessed with four items, including “I understand how my work contributes to my life's meaning”. The internal consistency of the scale was good (α = .85).

*Strengths use* was measured using four items of the strengths use scale [40], of which an example item is: “In my work I benefit from my strengths”. The scale had a seven-point response format, ranging from 1 (almost never) to 7 (almost always). The reliability analysis showed a strong internal consistency of the scale (α = .90).

*Work engagement* was measured with the validated nine-item Utrecht Work Engagement Scale (UWES; [41]). Example items are: “At work, I am bursting with energy” (vigor,), “I am enthusiastic about my job” (dedication), and “I am immersed in my work” (absorption). Participants used a seven-point frequency scale, ranging from (0) never to (6) always. The internal consistency of all three components of the UWES were adequate; vigor: α = .82, dedication: α = .90, absorption: α = .71.

*In-role Performance* was measured using the In-role Performance scale [42], which consists of seven items of which an example is: “Adequately completes assigned duties”. Participants had to score the items on a five-point scale ranging from (1) totally disagree to (5) totally agree. The reliability analysis showed a strong internal consistency of the scale (α = .91).

### Strategy of analysis

The meaningful work and performance model was tested with structural equation modelling (SEM) analyses using the AMOS software package [43]. The traditional chi-square, the goodness-of-fit index (GFI), and the root mean square error of approximation (RMSEA) were tested in order to assess the fit of the measurement model and the alternative models to the data. The incremental fit index (IFI), and the comparative fit index (CFI) were also assessed as recommended by Marsh, Balla and Hau [44]. The values of GFI, IFI, CFI > .90 and RMSEA < .08 indicate a reasonable fit of the model to the data [45,46]. The using of parcels in testing structural equation modelling result in more reliable measurement models [47]. We therefore conducted our SEM analysis on a partial disaggregation model [48] by creating parcels of items [49]. We created parcels of items for the variables ‘Meaningful work’, Strengths use’ and ‘In-role performance’, which were included in the model as latent factors with two indicators. ‘Work engagement’ was included as latent factor with the three abovementioned subscales as the indicators.

## Results

### Descriptive statistics

The means, standard deviations, reliabilities, and correlations, between all study variables are displayed in Table 1. In order to test the construct validity of the model variables (meaningful work, strengths use, work engagement, and performance), we tested a measurement model with the parcels tapping these latent variables. The measurement model showed a good fit to the data: χ^2^(18) = 864.85, *p* = .001; CFI = .992; TLI = .983; RMSEA = .051. All parcels had significant loadings on the intended factors (range λ = .69 − .97; *p* < .001). We could thus empirically distinguish between the various models.

**Table 1:** Means, standard deviations, correlations and cronbach's alphas.

	*M*	*SD*	1	2	3	4	5	6
1. Meaningful work	3.86	0.65	(.85)					
2. Strengths use	4.24	0.63	.66**	(.90)				
3. Vigor	4.75	1.04	.57**	.56**	(.82)			
4. Dedication	5.12	1.16	.73**	.68**	.79**	(.90)		
5. Absorption	4.64	1.01	.48**	.45**	.61**	.67**	(.71)	
6. Performance	5.85	0.86	.29**	.41**	.37**	.35**	.33**	(.91)

Note.* p < .05

** p < .01

### Test of the proposed model

**Fig 2:**
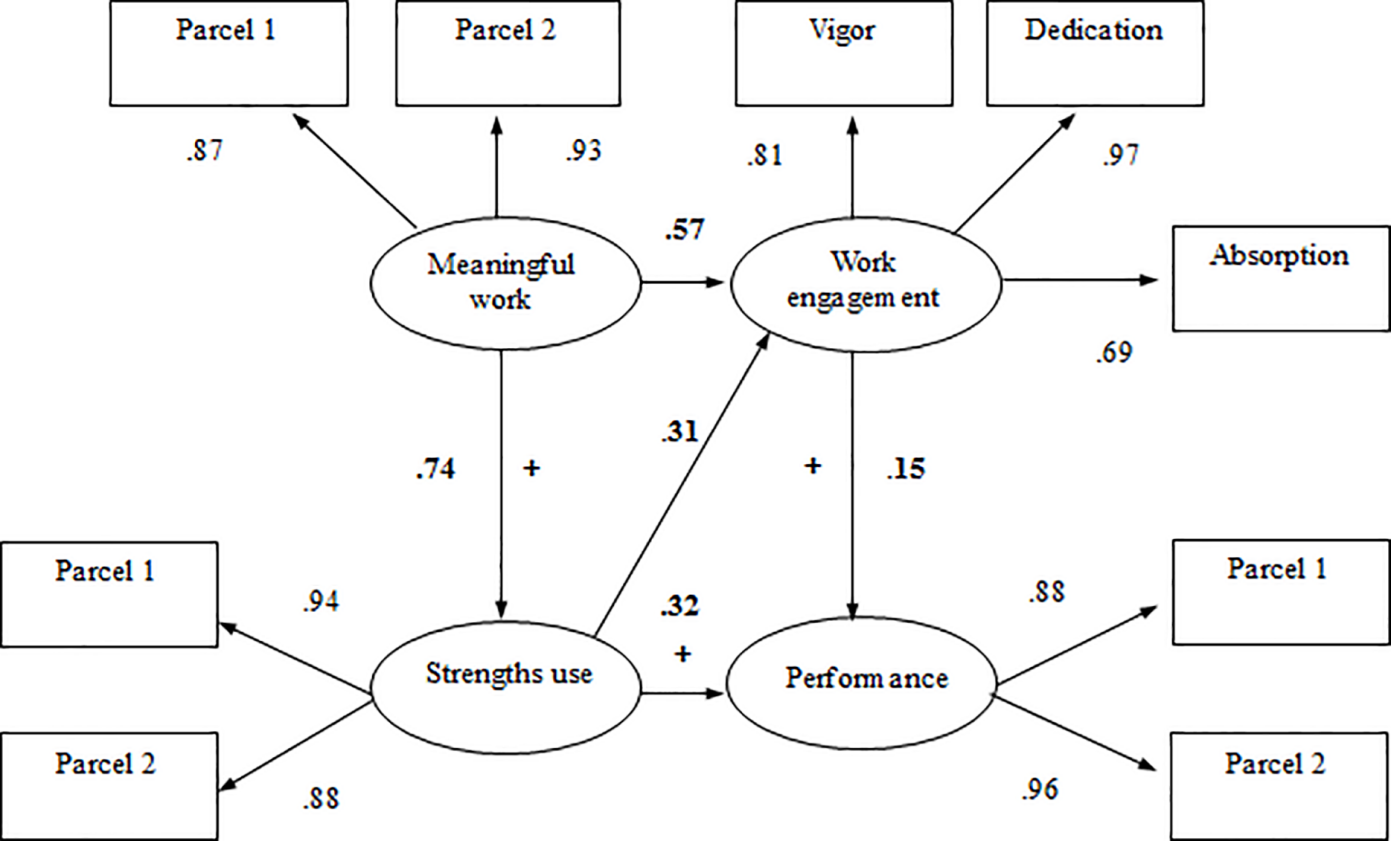
Maximum likelihood estimates for the meaningful work and performance model. Note. *N* = 459. All factor loadings and path coefficients are significant at the *p* < .001 level.

The results of the SEM-analyses indicated that the Proposed model (see Table 2 and Fig 2) fit well to the data: χ^2^(16) = 262.37, *p* = .001; CFI = .992; TLI = .983; RMSEA = .050. Meaningful work positively predicted both work engagement (β = .57, *p* < .001) and strengths use (β = .74, *p* < .001). Strengths use was positively related to both work engagement (β = .31, *p* < .001) and in-role performance (β = .32, *p* < .001). Lastly, work engagement also positively predicted in-role performance (β = .15, *p* = .036).

**Table 2:** Results of the SEM-analyses *(n = 459)*.

Model	*Χ*^*2*^	*df*	*Χ*^*2*^*/df*	CFI	TLI	IFI	RMSEA
Measurement model	864.85	18	48.05	.992	.983	.86	.051
Proposed model	262.37	16	16.40	.992	.983	.96	.050
Direct effects model	22.64	8	2.83	.776	.579	.99	.249
Sequential mediation model	156.48	24	6.52	.956	.918	.96	.110

We also tested two alternative models, namely a Sequential mediation model and a Direct effects model. The Sequential mediation model assumes a fully mediated link going from meaningful work to strength use, work engagement, and lastly in-role performance. The Direct effects model includes only the direct relationships between meaningful work, strengths use, and work engagement on the one hand, and in-role performance on the other.

The Sequential mediation model showed a bad fit to the data: χ^2^ (24) = 156.484, CFI = .956; TLI = .918; RMSEA = .110. The Proposed model fit significantly better to the data than the Sequential mediation model: Δχ^2^ (2) = 109.241, *p* < .001. The Direct effects model also showed a bad fit to the data: χ^2^ (8) = 22.64, CFI = .776; TLI = .579; RMSEA = .249. The Proposed model fit significantly better to the data than the Sequential mediation model: Δχ^2^ (2) = 654.317, *p* < .001.

## Discussion and conclusion

Results of this study demonstrate that meaningful work is related to performance in several and interrelated ways; via strengths use, via work engagement, and via strengths use affecting work engagement. This study supports our proposed meaningful work and performance model which accounts for these different pathways through which meaningful work affects performance. This demonstrates that the meaningful work performance relation is more complex than thought before [24]. Multiple factors–like strengths use and work engagement–mediate this relationship and determine the impact of meaningful work on performance. Results firmly highlight the impact of meaningful work within organizations.

### Theoretical contributions

This study advances our knowledge of the processes through which meaningful work affects performance at work. Previous studies have linked meaningful work to increased work engagement and performance [24], and to decreased burnout and increased employee well-being at work and at home [7]. This study has taken the impact of meaningful work to the next step by integrating multiple predictors into a comprehensive model. Our analysis demonstrates that the integral proposed model has a better fit than a sequential mediation model. This indicates that there are multiple pathways through which meaningful work affects performance. All in all, this endeavour has increased our understanding of the meaningful work–performance relationship within organizations.

This study further contributes to the growing body of research into strengths. Hitherto, our knowledge of the factors predicting strengths use at work was rather limited. Recent research has identified perceived strengths use support as a predictor of strength use telling that the subjective assessment of the support within the work environment to use your strengths affects whether or not employees use their strengths at work [34]. This is in line with the more general notion that subjective perception of work predicts behavior and attitude [35,36]. This same principle applies for the perception of meaningful work. When employees perceive their work as meaningful they are more likely to use their strengths. In this way this study contributes beyond the research on meaningful work and has implications for the growing body of research on strengths use as well.

### Limitations and avenues for future research

Although this study provides ample evidence for the hypothesized meaningful work—performance model, some limitations of this study need to be mentioned. First, the design of the study was cross-sectional and non-experimental. This is a shortcoming as this does not prove causality between the constructs under study. Longitudinal research designs are needed to determine this causality. Second, this study heavily relies on self-report measures susceptible to self-report bias. By using self-reports we cannot evade the common method bias, potentially increasing the correlations between the variables under study. Although self-reports might be the best way to accurately capture the subjective perceptions of employees [50], the results should be interpreted with care and future research might explore additional and more objective ways to measure the variables from this study [51]. Third, the results are based on data gathered in one organization operating in the healthy technology industry. Future research is necessary to test the meaningful work–performance model in other organizations, industries, and countries. Last, future research may also examine social components of meaningful work, both within the organization and influences from outside the organization (e.g. work socially (un)valued by society and/or work economically (un)valued).

Additional research is further required to explore other factors that potentially affect the proposed meaningful work–performance model. As meaningful work is an important predictor for performance, future research might consider other personal or job characteristics that stimulate the experience of meaningful work or the influence the meaningful work–performance relationship. For example, a closer look at the value fit between a person and the organization might be an interesting avenue for future research. This fit reflects the subjective fit between employee and the organization [52]. As the experience of meaningful work is more likely to occur when there is a strong connection between an employee and his or her work [16], this subjective fit might be a better predictor than more objective job characteristics. Future research might want to include other job characteristics that might influence the meaningful work–performance relationship. For example job characteristics mentioned within the job demands and resources theory, like autonomy or role clarity [37] might be relevant factors for the meaningful work–performance relationship.

### Practical implications and conclusion

The main practical implication of this study is that the meaningful work and performance are related in multiple ways. This makes the cultivation of meaningful work an important task for both management and HR. They could stimulate the perceptions of meaningful work, for example, by deliberately influencing how employees perceive their work and how the objectives of their work connect to their intrinsic values and beliefs. This could be done, by clearly communicating the goals, values, and practical contributions of the organization outside the organization. Especially, top management plays a crucial role in the cultivation of meaningful work within organizations by clearly communication the goals, values, and contributions of the organization. By initiating an ongoing dialogue within the organization employees will continuously reflect upon the meaningfulness of their work. All in all, this study provides practitioners with new ways to enhance performance within organizations.

In conclusion, this study has increased our understanding of the complex meaningful work and performance relationship. Although meaningful work affects performance in multiple ways, the necessity of meaningful work is often emphasized for other more ethical reasons. Some scholars consider experiencing meaningful work a human right and moral obligation from the employer [53]. Even more so, meaningful work operates as a buffer against burnout and adds to the well-being of employees [7]. Due to all these merits, we strongly advocate the deliberate cultivation of meaningful work within organizations. Therefore, we hope that this study motivates other researcher to further explore the meaningful work–performance relationship in order to increase both employee performance and employee well-being within contemporary organizations.

## Supporting information

S1 File 090518 PlosOne data motivational potential meaningful work.sav. (SAV)
